# Comparison of Structural and Functional Properties of Starches from the Rhizome and Bulbil of Chinese Yam (*Dioscorea opposita* Thunb.)

**DOI:** 10.3390/molecules23020427

**Published:** 2018-02-15

**Authors:** Biao Zhang, Ke Guo, Lingshang Lin, Cunxu Wei

**Affiliations:** 1 Key Laboratory of Crop Genetics and Physiology of Jiangsu Province/Key Laboratory of Plant Functional Genomics of the Ministry of Education, Yangzhou University, Yangzhou 225009, China; zhangbiao@yzu.edu.cn (B.Z.); 18115657147@163.com (K.G.); 18252713442@163.com (L.L.); 2 Co-Innovation Center for Modern Production Technology of Grain Crops of Jiangsu Province/Joint International Research Laboratory of Agriculture & Agri-Product Safety of the Ministry of Education, Yangzhou University, Yangzhou 225009, China

**Keywords:** Chinese yam, rhizome, bulbil, starch, structural and functional properties

## Abstract

Chinese yam is an important edible starch plant and widely cultivated in China. Its rhizome and bulbil are starch storage tissues below and above ground, respectively. In this paper, starches were isolated from the rhizome and bulbil of Chinese yam, and their structural and functional properties were compared. Both starches had an oval shape with an eccentric hilum and a C_A_-type crystalline structure. Their short-range ordered structure and lamellar structure had no significant difference. However, the rhizome starch had a significantly bigger granule size and lower amylose content than the bulbil starch. The swelling power and water solubility were significantly lower in the rhizome starch than in the bulbil starch. The onset and peak gelatinization temperatures were significantly higher in the rhizome starch than in the bulbil starch. The rhizome starch had a significantly higher breakdown viscosity and a lower setback viscosity than the bulbil starch. The thermal stability was lower in the rhizome starch than in the bulbil starch. The rhizome starch had a significantly lower resistance to hydrolysis and in vitro digestion than the bulbil starch. The above results provide important information for the utilization of rhizome and bulbil starches of Chinese yam.

## 1. Introduction

Starch consists mainly of linear amylose and highly branched amylopectin and is stored as semicrystalline granules [1]. Starch not only is used as food to provide nutrition for humans and animals, but also is widely used as an important ingredient in the food, textile, medicine, papermaking, casting, metallurgy, petroleum, and chemical industries [2]. The diversified applicability of starch demands specific structural and functional properties. Starches from different plant sources are varied in their morphologies, structures, and properties, which determine their utilization [3,4]. Some studies have reported the structural and functional properties of starches from commercial important sources, such as cereals, tubers, and legumes [5,6,7]. In recent years, in order to save food, broaden the source of starches, and develop new functional starches, substantial efforts have been made to find starches from non-conventional sources and study their structural and functional properties [2,8,9,10,11].

Yam is a generic name for the plants of twining climbers that form tubers or rhizomes in the genus *Dioscorea* of the monocot family Dioscoreaceae. World production of yams reached over 65.9 million metric tons in 2016, and the top three producers are Nigeria, Côte d’Ivoire, and Ghana with production quantities over 31.5, 4.9, and 4.5 million metric tons, respectively, every year from 1997 to 2016 [12]. Chinese yam (*Dioscorea opposite* Thunb.) is a rhizome crop mainly cultivated in China, Japan, and Korea [13]. The rhizome of Chinese yam is an important edible and pharmaceutical food in China, and has nutritional and economic significance [14,15,16,17]. Starch is the main component of rhizome. Its structural and functional properties have been studied by some previous reports [15,16,17,18]. The bulbil, also known as Shanyaodou (in Chinese), is an aerial tuber of Chinese yam, which is generated from leaf axil [2,19]. The bulbil can be released from the parent plant and grow independently into a new plant, making it an important propagation organ [13,19]. The bulbil of Chinese yam has been used as an important food in people’s everyday life and as an important ingredient in livestock feed. It has also been used as one of the important ingredients for invigorating the spleen and stomach, promoting the production of body fluids, benefiting the lung, and invigorating the kidney [2,13,20]. The yield of bulbils of Chinese yam is from 3000 to 4500 kg dry weight per hectare. Starch is the main component in bulbil and can account for about 60% in dry bulbil [2,13,20]. However, the structural and functional properties of starch from bulbil have not been studied but there has been one previous study investigating its physicochemical properties [2]. It is of great importance to understand the structural and functional properties of rhizome and bulbil starches from the same Chinese yam cultivar.

In this study, starches were isolated from rhizomes and bulbils of Chinese yam cultivar Shuishanyao. Their morphology, granule size, amylose content, crystallinity, short-range ordered structure, lamellar structure, swelling power, water solubility, thermal and pasting properties, thermal stability, and hydrolysis and digestion properties were investigated using many physicochemical measuring methods and spectral analysis machines. Our objective was to compare the structural and functional properties of rhizome and bulbil starches and provide some information as to how these starches can be used.

## 2. Results and Discussion

### 2.1. Soluble Sugar and Starch Contents in Rhizome and Bulbil

The soluble sugar and starch contents in dry rhizome and bulbil are presented in Table 1. The bulbil had significantly higher soluble sugar and lower starch than the rhizome. The 60.5% starch content in dry bulbil and about 20% starch content in fresh bulbil of Chinese yam that were reported in [2] are similar to the present result. The aboveground bulbil has lower starch content and higher soluble sugar content than the underground bulb in the same plant of *Lilium lancifolium* [21]. The high starch content in the rhizome and bulbil of Chinese yam indicated that both are good starch sources.

### 2.2. Morphology and Granule Size Distribution of Starches from Rhizome and Bulbil

**Figure 1:**
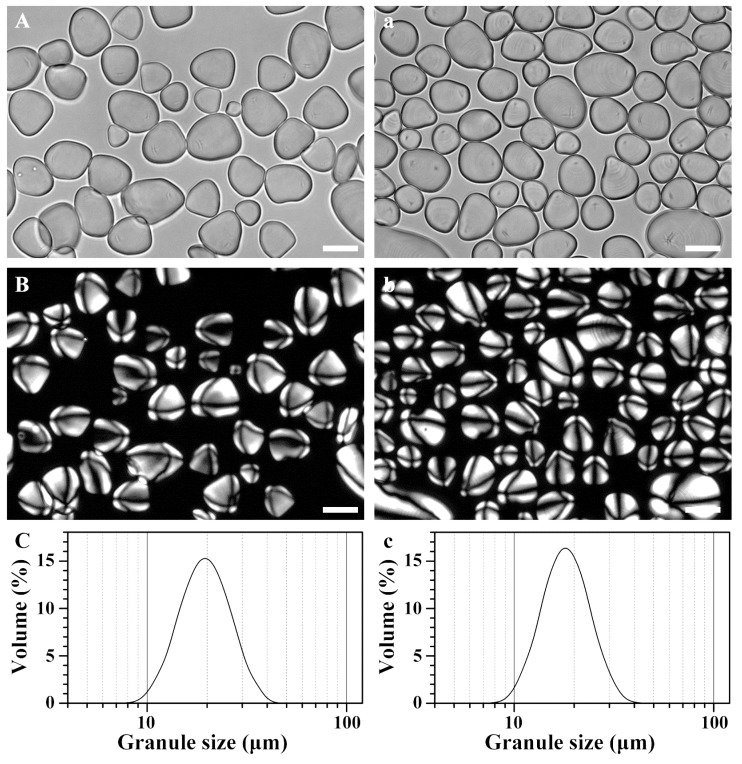
Morphology under normal light (**A**,**a**) and polarized light (**B**,**b**) and size distribution (**C**,**c**) of starch granules from rhizome (**A**–**C**) and bulbil (**a**–**c**) of Chinese yam. Scale bar = 20 μm.

The isolated starch granules were observed under normal and polarized light. The starches from rhizome and bulbil were similar and exhibited an oval shape with the hilum at one end of the granule (Figure 1). The present result agreed with the previous report [2,22]. Native starch granules have a semicrystalline structure, known as the typical Maltese cross, which can be observed under a polarized light microscope. Starch granules with centric or eccentric hila have been reported in different plants and may be related to the different botany origin [22]. The granule size of the starch was analyzed by a laser diffraction particle size analyzer. The result showed that both rhizome and bulbil starches showed a unimodal size distribution ranging from 9 to 40 μm (Figure 1). The mean diameter of starch granules is listed in Table 2. The bulbil starch granules were smaller than the rhizome starch granules. A similar result has been reported in the bulbil and bulb of *Lilium lancifolium* [21]. Starch granule size can be affected by amyloplast development, plant physiology, and other environmental factors [23].

### 2.3. Amylose Content in Starches from Rhizome and Bulbil

The rhizome and bulbil starch contained 35.2% and 38.3% amylose content, respectively (Table 2), which was higher than normal potato starch with 20–31% amylose content, maize starch with 22–33% amylose content, rice starch with 5–28% amylose content, and wheat starch with 18–30% amylose content [5,24]. Zhou et al. [2] reported that the amylose content of bulbil starch ranged from 33.3% to 36.7% among different Chinese yam cultivars. Amylose content is an important factor affecting starch structure and properties, which determine the applications of starch and the characteristics of final products.

### 2.4. Crystalline Structure of Starches from Rhizome and Bulbil

**Figure 2:**
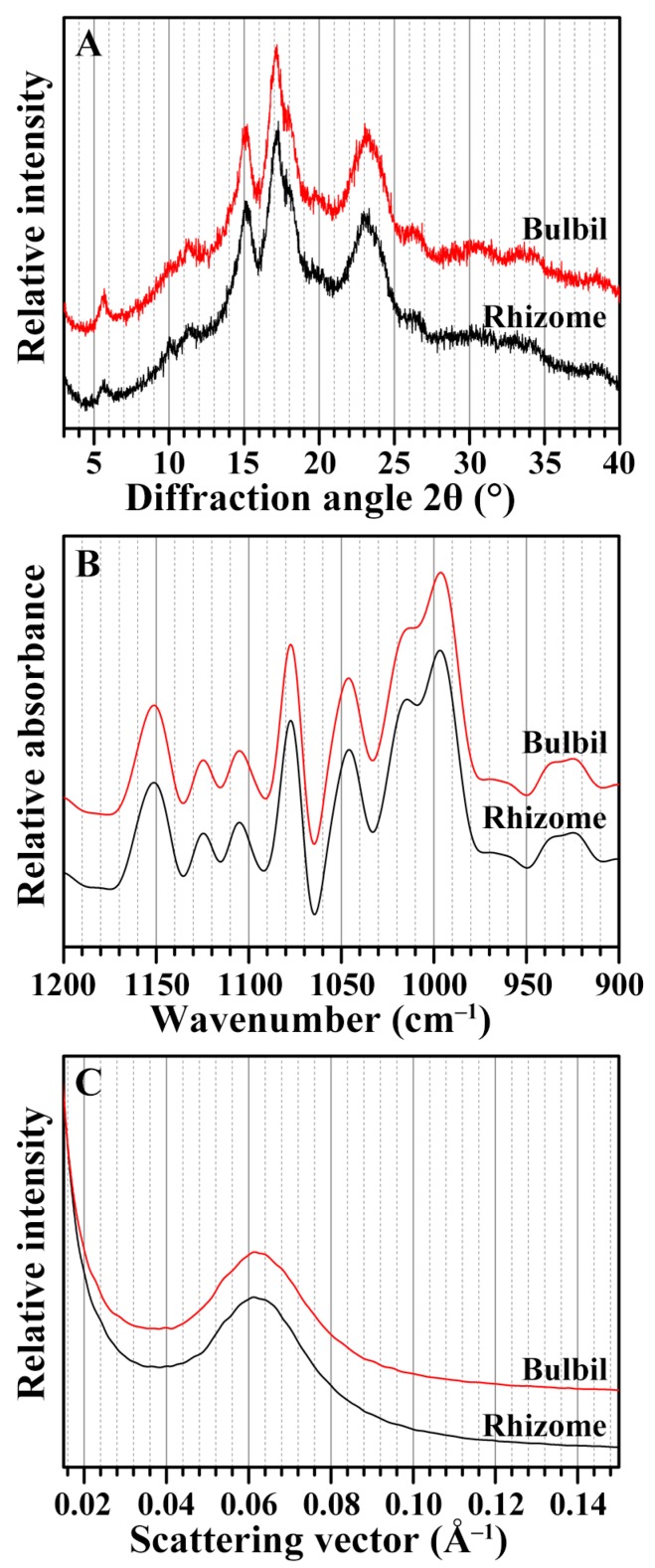
XRD pattern (**A**); FTIR spectrum (**B**); and SAXS profile (**C**) of starches from the rhizome and the bulbil of Chinese yam.

Starches from different plant sources can be divided into A-, B-, and C-type according to their X-ray diffraction (XRD) patterns. Usually, normal cereal seeds have A-type starch, most tubers contain B-type starch, and some rhizomes and leguminous seeds have C-type starch [25]. The XRD patterns of the rhizome and bulbil starches are shown in Figure 2A. They showed four reflection peaks at about 5.6°, 15°, 17°, and 23° 2θ, indicating that both rhizome starch and bulbil starch are C-type starches. The relative crystallinity was similar in both rhizome and bulbil starches (Table 3). The C-type starch in the rhizome and bulbil of Chinese yam has been reported in previous literature [2,15,16,17]. The C-type starch contains A- and B-type crystallinity, and can be further divided into C_A_-(closer to A-type), C_C_-(typical C-type), and C_B_-(closer to B-type) type according to the proportion of A- and B-type crystallinity. The C_A_-type starch has a shoulder peak at 18° 2θ, and C_B_-type starch has two shoulder peaks at 22° and 24° 2θ [25]. According to the above, the XRD patterns of the rhizome and bulbil starches had a shoulder peak at 18° 2θ, indicating that they were C_A_-type starch. C_A_-, C_C_-, and C_B_-type starches have been reported in bulbils of different Chinese yam cultivars [2]. The proportion of A- and B-type crystallinity in C-type starch could be affected by its growing environment.

### 2.5. Short-Range Ordered Structure of Starches from Rhizome and Bulbil

The short-range ordered structure of starches, defined as the double-helical order, can be detected using a Fourier transform infrared (FTIR) spectrometer. The bands at 1045 and 1022 cm^−1^ are associated with ordered/crystalline and amorphous regions in starch, respectively. The ratio of absorbance, 1045/1022 cm^−1^, can be used to quantify the ordered degree, and that of 1022/995 cm^−1^ is used to measure the proportion of amorphous to ordered carbohydrate structure in the starch [26]. The FTIR spectra of both rhizome and bulbil starches of Chinese yam are presented in Figure 2B, and their ratios of 1045/1022 and 1022/995 cm^−1^ are shown in Table 3. The results showed that both rhizome and bulbil starches had a similar short-range ordered structure, which may be attributed to their similar crystalline structure. Starches from the bulbil and the bulb of *Lilium lancifolium* also present similar IR ratios at 1045/1022 and 1022/995 cm^−1^ [21], but starches from the seed and the rhizome of lotus have significantly different IR ratios at 1045/1022 and 1022/995 cm^−1^ [27]. The different ordered structures of starch might be related to botany origin.

### 2.6. Lamellar Structure of Starches from Rhizome and Bulbil

The lamellar structure of starch can be analyzed using small-angle X-ray scattering (SAXS) spectra. The SAXS spectra of the rhizome and bulbil starches are shown in Figure 2C, and their parameters are listed in Table 3. The scattering peak position arises from the periodic arrangement of alternating crystalline and amorphous lamellae of amylopectin, and corresponds to the lamellar repeat distance or Bragg spacing. The location of the peak depends on the size of lamella, and may differ among starches from different plants. The peak intensity results from the electron density difference between the crystalline and amorphous regions of the lamellae [28]. The scattering peak position, Bragg spacing, and peak intensity were similar between both rhizome and bulbil starches, indicating that the lamellar structure of the starch is not different between rhizome and bulbil, which was in agreement with the results of XRD and FTIR.

### 2.7. Swelling Power and Water Solubility of Starches from Rhizome and Bulbil

**Figure 3:**
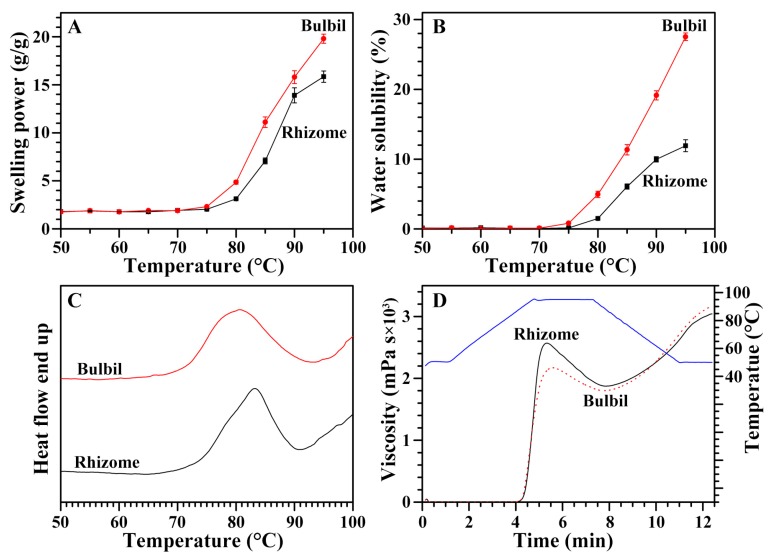
Swelling power (**A**); water solubility (**B**); DSC thermogram (**C**); and RVA profile (**D**) of starches from the rhizome and the bulbil of Chinese yam.

The swelling power and water solubility of both rhizome and bulbil starches are shown in Figure 3A,B. Before 75 °C, swelling power and water solubility were not detected in both rhizome and bulbil starches. After 75 °C, swelling power and water solubility gradually increased with increasing heating temperature. The swelling power and the water solubility were significantly higher in the bulbil starch than in the rhizome starch. The swelling power of starch indicates the degree of water absorption of a starch granule, and the water solubility reflects the degree of dissolution during the starch swelling procedure [29]. The hydration and swelling of starch during heating reflect the magnitude of interaction between starch chains within the amorphous and crystalline domains. The extent of this interaction is influenced by the granule morphology and size, amylose content, and crystalline structure [11]. In the present study, the differences in swelling power and solubility between the rhizome and bulbil starches might be due to the differences in granule size and amylose content.

### 2.8. Thermal Properties of Starches from Rhizome and Bulbil

The thermal properties of both rhizome starch and the bulbil starch were analyzed by a differential scanning calorimeter (DSC), and their thermograms and thermal parameters are given in Figure 3C and Table 4. The bulbil starch had a significantly lower gelatinization onset and peak temperature than the rhizome starch, but its gelatinization temperature range was larger than that of the rhizome starch. The gelatinization conclusion temperature and enthalpy were similar between the rhizome and bulbil starches. The thermal properties of starch are affected by factors such as granule size, amylose content, and crystalline structure [30].

### 2.9. Pasting Properties of Starches from Rhizome and Bulbil

The pasting properties of starch were determined by a rapid visco analyzer (RVA), and their RVA patterns and parameters are given in Figure 3D and Table 5. The hot viscosity, final viscosity, and pasting temperature were similar between the rhizome and bulbil starches, but the bulbil starch had significantly lower peak and breakdown viscosities and a higher setback viscosity and peak time than rhizome starch. The breakdown viscosity reflects paste stability, and setback viscosity shows the gelling ability or retrogradation tendency of starch [31,32]. Pasting properties are influenced by the size and shape of the starch granules, the amylose content, and the branch-chain length distribution of amylopectin. The amylose leached from swollen granules during heating is re-associated to form a network during the cooling process and increase the final viscosity [33]. The different pasting properties between the rhizome and bulbil starches might result from the different granule size, amylose content, and swelling power [34].

### 2.10. Thermal Stability of Starches from Rhizome and Bulbil

**Figure 4:**
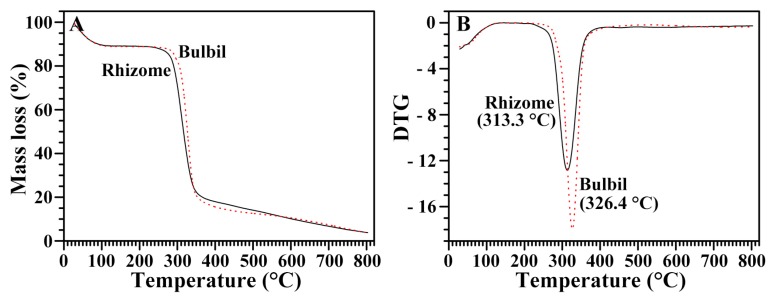
Thermogravimetric (**A**) and derivative thermogravimetric (DTG); (**B**) curves of starches from the rhizome and the bulbil of Chinese yam.

The thermogravimetric analysis (TGA) curves of the rhizome and bulbil starches are presented in Figure 4A. Two well-defined shifts were clearly detected. There are two crystal structures: one is from the ordered packing between starch molecular chains by the interaction of hydrogen bonds, and the other is from (between) the ordered packing including starch molecular chains and water molecules. The first shift of a TGA curve is produced by water evaporation in starch, and corresponds with the destruction of the starch-water structure. The second shift is the decomposition of the starch, and is related to the destruction of the starch-starch structure [35]. Derivative thermogravimetric (DTG) curves were performed in order to identify the temperatures at which the maximum thermal degradation rates of each component occurred (Figure 4B). The decomposition temperature reflects the maximum rate of mass loss, and occurred at 313.3 °C in the rhizome starch and at 326.4 °C in the bulbil starch, indicating that the thermal stability was higher in the bulbil starch than in the rhizome starch.

### 2.11. Hydrolysis of Starches from Rhizome and Bulbil

**Figure 5:**
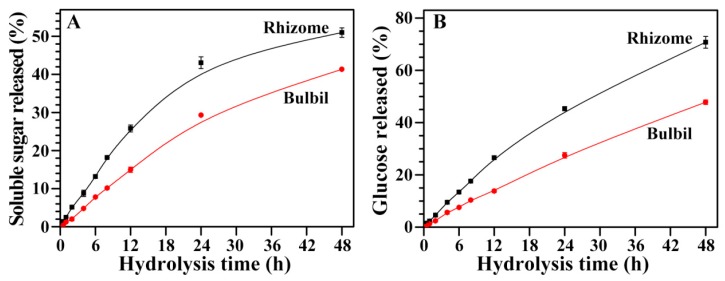
Hydrolysis curve by porcine pancreatic (PPA) (**A**) and in vitro digestion curve by both PPA and amyloglucosidase from *Aspergillus niger* (AAG) (**B**) of starches from the rhizome and the bulbil of Chinese yam.

The rhizome and bulbil starches were hydrolyzed with α-amylase from porcine pancreatic (PPA) by measuring the released soluble carbohydrate (Figure 5A). The hydrolysis kinetics was similar between the rhizome and bulbil starches, but the bulbil starch had significantly higher resistance than the rhizome starch. PPA pits the starch granule surface first, then penetrates into the interior and hydrolyzes the granule from the inside out [36]. The susceptibility of starch granules to PPA attack is affected by granule morphology, size, integrity, porosity, amylose content, and crystalline structure [37]. The amylose content is inversely related to starch hydrolysis by amylase [36]. In the present study, the slow hydrolysis of the bulbil starch by PPA might result from its high amylose content compared with the rhizome starch.

### 2.12. In Vitro Digestion Properties of Starches from Rhizome and Bulbil

Starch digestion in the human body is typically viewed as a sequential reaction beginning with α-amylase and followed by mucosal α-glucosidase to produce glucose [38]. As a biochemical mimic of in vivo conditions, an in vitro study of starch digestion is normally carried out using two kinds of enzyme: PPA and fungal amyloglucosidase [39]. The in vitro kinetic digestion degrees of the rhizome and bulbil starches were analyzed by determining the released glucose at different times during digestion with both PPA and amyloglucosidase from *Aspergillus niger* (AAG) (Figure 5B). During digestion, bulbil starch had a higher resistance to hydrolysis than rhizome starch. The differences in the in vitro digestibility among different starches result from the interplay of many factors, such as starch source, morphology, granule size, amylose content, and crystalline structure [40]. Usually, the size and amylose content are negatively correlated with the digestion of native starch [36,41]. In the present study, the rhizome and bulbil starches had a similar morphology and crystalline structure, including crystallinity, short-range ordered structure, and lamellar structure, but the amylose content was significantly higher in the bulbil starch than in the rhizome starch. Therefore, the high amylose content in bulbil starch might increase its resistance to in vitro digestion compared with rhizome starch.

## 3. Materials and Methods

### 3.1. Plant Materials

The fresh rhizome and bulbil of Chinese yam (*Dioscorea opposita* Thunb. cultivar Shuishanyao) were obtained from a local natural food market (Yangzhou City, China) in December 2016.

### 3.2. Measurements of Soluble Sugar and Starch Contents in Rhizome and Bulbil

The rhizomes and bulbils were sliced into small pieces and dried in an oven at 110 °C for 2 h and 80 °C for 2 days, and then ground extensively. The flour was passed through a 100-mesh sieve, and its starch and soluble sugar contents were determined using the colorimetric method of anthrone-H_2_SO_4_ as previously described [42].

### 3.3. Isolation of Starch

Rhizomes were peeled and sliced into small pieces, and bulbils were cleanly washed. The samples were homogenized with deionized water and filtered with 100- and 200-mesh sieves. The starch suspension was settled for 6 h. The precipitated white starch was treated three times with 0.2% NaOH to remove surface protein and mucopolysaccharide. The treated starch was washed three times with deionized water and two times with anhydrous ethanol, dried at 40 °C for 2 days, and ground and passed through a 100-mesh sieve.

### 3.4. Morphology Observation and Size Analysis of Starch

The morphology of starch under normal light and polarized light was observed using a BX53 polarizing light microscope (Olympus, Tokyo, Japan) equipped with a charge-coupled device (CCD) camera (DP72, Olympus, Tokyo, Japan) as previously described [22]. The granule size distribution of starch was analyzed using a laser diffraction particle size analyzer (Mastersizer 2000, Malvern, UK) as previously described [43].

### 3.5. Measurement of Amylose Content in Starch

The amylose content was determined using the iodine colorimetric method as previously described [44].

### 3.6. Analysis of Crystalline Structure of Starch

The crystalline structure of starch was analyzed using an X-ray powder diffractometer (D8, Bruker, Karlsruhe, Germany) at 200 mA and 40 kV as previously described [43]. The scanning region of diffraction angle was from 3 to 40° 2θ with a step size of 0.02° and a count time of 0.8 s.

### 3.7. Analysis of Short-Range Ordered Structure of Starch

The short-range ordered structure of starch was analyzed using a Varian 7000 FTIR spectrometer with a DTGS detector equipped with an attenuated total reflectance single-reflectance cell containing a germanium crystal (45° incidence angle) (PIKE Technologies, Madison, WI, USA) as previously described [43].

### 3.8. Determination of Swelling Power and Water Solubility of Starch

The swelling power and water solubility of starch were determined by heating starch-water slurries at temperatures from 50 to 95 °C in 5 °C intervals as previously described [43].

### 3.9. Analysis of Thermal Properties of Starch

The thermal properties of starch were measured by a DSC (200-F3, NETZSCH, Selb, Germany) as previously described [44].

### 3.10. Analysis of Pasting Properties of Starch

The pasting properties of starch (8% solids) were analyzed using an RVA (3D, Newport Scientific, Warriewood, NSW, Australia) following the temperature program: holding at 50 °C for 1 min, heating to 95 °C at 12 °C/min, maintaining at 95 °C for 2.5 min, cooling to 50 °C at 12 °C/min, and holding at 50 °C for 1.4 min.

### 3.11. Thermogravimetric Analysis of Starch

Ten milligrams of starch were placed on a platinum pan and heated from room temperature to 800 °C using a Pyris 1 TGA system (PerkinElmer, Waltham, MA, USA) at a heating rate of 10 °C/min. Nitrogen was used as a purge gas at a flow rate of 20 mL/min. Change in sample weight against temperature was measured.

### 3.12. Measurement of PPA Hydrolysis of Starch

The starch was hydrolyzed by PPA (A3176, Sigma Aldrich, St Louis, MO, USA) for different times as previously described [42]. After hydrolysis, starch slurries were quickly centrifuged at 5000 *g* for 5 min. The supernatant was used for the measurement of the solubilized carbohydrates to quantify the degree of hydrolysis using the anthrone-H_2_SO_4_ method.

### 3.13. Analysis of In Vitro Digestion of Starch

The in vitro digestion of starch was determined using both PPA (Sigma A3176) and AAG (E-AMGDF, Megazyme, Bray, Ireland) as previously described [44]. The released glucose quantity was determined using a glucose assay kit (K-GLIC, Megazyme, Bray, Ireland).

### 3.14. Statistical Analysis

The mean value and significant difference were analyzed using SPSS software (IBM Company, Chicago, IL, USA).

## 4. Conclusions

The bulbil and the rhizome of Chinese yam had 66.9% and 84.6% starch content, respectively. Their starches were oval in shape and had eccentric hila. The volume- and surface-weighted mean diameters were 16.4 and 8.5 μm for the bulbil starch, respectively, and 17.7 and 9.4 μm for the rhizome starch, respectively. The bulbil starch and the rhizome starch had 38.3% and 35.2% amylose content, respectively. Both rhizome and bulbil starches had C_A_-type crystallinity and showed a similar short-range ordered structure and lamellar structure. The rhizome starch had lower swelling power, water solubility, and setback viscosity and higher onset and peak gelatinization temperatures and breakdown viscosity than the bulbil starch. The bulbil starch had higher thermal stability, resistance to hydrolysis, and in vitro digestion than the rhizome starch.

## Figures and Tables

**Table 1:** Soluble sugar and starch contents in dry rhizome and bulbil ^a^.

Tissues	Soluble Sugar Content (%db)	Starch Content (%db)
Rhizome	1.1 ± 0.1	84.6 ± 1.8
Bulbil	1.7 ± 0.1 **	66.9 ± 2.9 **

^a^ Data are the means ± standard deviations, *n* = 3. * The bulbil data are significantly different compared with the rhizome data (** for *p* < 0.01).

**Table 2:** Granule size distribution and amylose content of starches ^a^.

Tissues	Size Distribution ^b^					Amylose Content (%)
	D[4,3] (μm)	D[3,2] (μm)	d(0.1) (μm)	d(0.5) (μm)	d(0.9) (μm)	
Rhizome	17.74 ± 0.02	9.36 ± 0.01	10.51 ± 0.01	17.81 ± 0.02	26.03 ± 0.03	35.2 ± 0.5
Bulbil	16.42 ± 0.01 ***	8.48 ± 0.01 ***	10.17 ± 0.01 ***	16.58 ± 0.01 ***	23.64 ± 0.01 ***	38.3 ± 0.1 **

^a^ Data are the means ± standard deviations, *n* = 3. ^b^ D[4,3] and D[3,2] are the volume- and surface-weighted mean diameters, respectively. d(0.1), d(0.5), and d(0.9) are the granule size at which 10%, 50%, and 90% of all of the granules by volume are smaller. * The bulbil data are significantly different compared with the rhizome data (** for *p* < 0.01 and *** for *p* < 0.001).

**Table 3:** Relative crystallinity, IR ratio, and lamellar structure parameters of starches ^a^.

Tissues	Relative Crystallinity (%)	IR Ratio (cm^−1^)		Lamellar Structure Parameters ^b^		
		1045/1022	1022/995	*S*_max_ (Å^−1^)	*D* (nm)	*I*_max_ (Counts)
Rhizome	27.9 ± 1.8	0.71 ± 0.01	0.75 ± 0.04	0.062 ± 0.001	10.15 ± 0.08	180.7 ± 16.7
Bulbil	26.8 ± 1.8	0.70 ± 0.01	0.76 ± 0.01	0.062 ± 0.001	10.03 ± 0.20	161.4 ± 9.5

^a^ Data are the means ± standard deviations, *n* = 2. ^b^ *S*_max_, peak position; *D*, Bragg spacing; *I*_max_, peak intensity.

**Table 4:** Thermal parameters of starches ^a^.

Tissues	T_o_ (°C) ^b^	T_p_ (°C) ^b^	T_c_ (°C) ^b^	ΔT (°C) ^b^	ΔH (J/g) ^b^
Rhizome	73.6 ± 0.5	83.1 ± 0.1	88.6 ± 0.5	15.0 ± 0.9	12.3 ± 0.6
Bulbil	71.7 ± 0.3 **	81.1 ± 0.4 **	89.2 ± 0.8	17.5 ± 0.6 *	13.0 ± 0.2

^a^ Data are the means ± standard deviations, *n* = 3. ^b^ T_o_, onset temperature; T_p_, peak temperature; T_c_, conclusion temperature; ΔT, gelatinization temperature range (T_c_–T_o_); ΔH, gelatinization enthalpy. * The data bulbil data are significantly different compared with the rhizome data (* for *p* < 0.05 and ** for *p* < 0.01).

**Table 5:** Pasting properties of starches ^a^.

Tissues	PV (mPa s) ^b^	HV (mPa s) ^b^	BV (mPa s) ^b^	FV (mPa s) ^b^	SV (mPa s) ^b^	PT (min) ^b^	P_Temp_ (°C) ^b^
Rhizome	5145 ± 108	3752 ± 171	1393 ± 63	6065 ± 179	2313 ± 20	5.36 ± 0.04	88.7 ± 0.1
Bulbil	4288 ± 48 ***	3542 ± 53	747 ± 14 ***	6293 ± 37	2751 ± 27 ***	5.58 ± 0.04 **	88.4 ± 0.4

^a^ Data are the means ± standard deviations, *n* = 3. ^b^ PV, peak viscosity; HV, hot viscosity; BV, breakdown viscosity (PV–HV); FV, final viscosity; SV, setback viscosity (FV–HV); PT, peak time; P_Temp_, pasting temperature. * The data bulbil data are significantly different compared with the rhizome data (** for *p* < 0.01 and *** for *p* < 0.001).
